# Astragaloside and/or Hydroxysafflor Yellow A Attenuates Oxygen-Glucose Deprivation-Induced Cultured Brain Microvessel Endothelial Cell Death through Downregulation of PHLPP-1

**DOI:** 10.1155/2020/3597527

**Published:** 2020-12-15

**Authors:** Jinyi Cao, Kai Wang, Lu Lei, Lu Bai, Ruimin Liang, Yi Qiao, Jialin Duan, Kai Gao, Shanshan Cao, Chao Zhao, Zhifu Yang

**Affiliations:** ^1^Department of Pharmacy, Xijing Hospital, Fourth Military Medical University, Xi'an 710032, China; ^2^Department of Pharmacy, Xi'an No. 1 Hospital, Xi'an 710002, China

## Abstract

The incidence of ischemic stroke, a life-threatening condition in humans, amongst Asians is high and the prognosis is poor. In the absence of effective therapeutics, traditional Chinese medicines have been used that have shown promising results. It is crucial to identify traditional Chinese medicine formulas that protect the blood-brain barrier, which is damaged by an ischemic stroke. In this study, we aimed to elucidate such formulas. Brain microvascular endothelial cells (BMECs) were used to establish an *in vitro* ischemia-reperfusion model for oxygen-glucose deprivation (OGD) experiments to evaluate the function of two traditional Chinese medicines, namely, astragaloside (AS-IV) and hydroxysafflor yellow A (HSYA), in protecting against BMEC. Our results revealed that AS-IV and HSYA attenuated the cell loss caused by OGD by increasing cell proliferation and inhibiting cell apoptosis. In addition, these compounds promoted the migration and invasion of BMECs *in vitro*. Furthermore, we found that BMECs rescued by AS-IV and HSYA could be functionally activated *in vitro*, with AS-IV and HSYA showing synergetic effects in rescuing BMECs survival *in vitro* by reducing the expression of PHLPP-1 and activating Akt signaling. Our results elucidated the potential of AS-IV and HSYA in the prevention and treatment of stroke by protecting against cerebral ischemia-reperfusion injury.

## 1. Introduction

Globally, over 15 million people are known to suffer from stroke annually. Although the treatment strategies for stroke have dramatically improved, it still ranks as the second leading cause of mortality and disability [1]. Recent reports indicate that other than the high stroke mortality in Asian countries compared with that in other countries, the effects are higher amongst Asians and include severe intracerebral hemorrhages [2]. Presently, there are no effective pharmacological therapies for the treatment of stroke [3, 4]. Ischemic stroke accounts for more than 85% of all stroke cases, resulting in severe acute necrosis and neuronal cell apoptosis [5]. Moreover, stroke damages the blood-brain barrier (BBB), which protects the central nervous system. Several reports have shown that BBB dysfunction is associated with multiple neurological disorders and that its integrity is critical for normal neuronal function [6, 7]. The integrity of the BBB primarily depends on a population of cells called brain microvascular endothelial cells (BMECs) [8, 9]. Thus, it is crucial to identify drugs that protect BMECs against ischemia-reperfusion injury and promote their recovery after an ischemic incident.

For over 2000 years, traditional Chinese medicines have been studied and used in China. Their use in treating patients with cerebrovascular disease dates back to the Han dynasty (202 B. C.–220 A. D.). Moreover, certain reports state the use of some of its formulas for stroke treatment and recovery [10]. For example, astragaloside (AS-IV), extracted from Huang Qi (Radix Astragali Seu Hedysari), is a traditional Chinese medicine most popularly used in cerebrovascular diseases. Its molecular mechanisms have been studied for over two decades [11, 12], with multiple studies in rats and mice showing that it exerts protective effects during ischemia-reperfusion injury by regulating inflammation and cell death via several signaling pathways [13–18]. Moreover, data from an *in situ* study in rats with ischemia-reperfusion injury showed that it attenuated increased BBB permeability [18], suggesting its promising role in maintaining the integrity of the BBB. Hydroxysafflor yellow A (HSYA) is another traditional Chinese medicine with wide applications in the treatment of stroke. Interestingly, it is also an Iranian folklore medicine used to treat cerebral ischemia-reperfusion injury [19]. HSYA has long been known to protect against cerebral ischemia-reperfusion injury *in vivo* through its antioxidant activities [20]. Multiple studies in rats, mice, and rabbits have demonstrated that it protects against ischemia-reperfusion injury by regulating toll-like receptors (TLRs), extracellular signal-regulated kinase (ERK) signaling, and protein kinase B (Akt) signaling pathways [21–30]. However, the effects of AS-IV and HSYA need to be further elucidated, especially their ability to protect BMECs from ischemia-reperfusion injury. Moreover, although their combination has been used to promote the recovery of stroke patients, it is still unclear whether the combination therapy is more efficacious in protecting BMECs and recovering from a stroke.

We used BMECs to establish an *in vitro* ischemia-reperfusion model for oxygen-glucose deprivation/recovery (OGD/R) experiments to evaluate the effect of AS-IV and HSYA on BMECs during OGD. Furthermore, we determined the viability and proliferation of BMECs during treatment and the role of these drugs in apoptosis and migration in OGD.

## 2. Materials and Methods

### 2.1. Astragaloside IV Concentration in Huang Qi Injection

The concentration of AS-IV in Huang Qi injection (HQI) was determined using a liquid chromatographic system from Shimadzu Instruments (Kyoto, Japan). The system consisted of an LC-10AVP binary pump and an evaporative light-scattering detector, connected to a computer system (for data acquisition) from LC-Solution. The analytical column used was the Agilent Zorbax Extend C18 column (250 mm × 4.6 mm, 5 *μ*m) from Agilent Corporation. The mobile phase was a mixture of acetonitrile and water in a ratio of 36 : 64 at a flow rate of 1.0 mL/min. The injection volume, detector temperature, and pressure were 10 *μ*L, 40°C, and 0.35 MPa, respectively.

### 2.2. Hydroxysafflor Yellow A Concentration in Honghua Injection

Analyses were performed using the Agilent 1100 series system from Agilent Corporation (USA), coupled with a diode-array detector. The analytical column used was the Agilent Zorbax Extend C18 column (250 mm × 4.6 mm, 5 *μ*m) from Agilent Corporation. The mobile phase was composed of a mixture of acetonitrile and 0.02 M NaH_2_PO_4_ (adjusted to pH 3.5 with orthophosphoric acid). Gradient elution was performed as follows: initial (0 min), 10% acetonitrile and 0.02 M NaH_2_PO_4_; 0 to 16 min, linear increase from 10 to 22% acetonitrile and 0.02 M NaH_2_PO_4_. The system was balanced for 5 min using the initial mobile phase (acetonitrile and 0.02 M NaH_2_PO_4_/orthophosphoric acid = 1/9), after detecting each sample. The flow rate, injection volume, and detection wavelength were 0.8 mL/min, 10 *μ*L, and 403 nm, respectively.

### 2.3. BMEC Cultures, OGD, and Drug Treatments

Mouse primary BMECs were purchased from Cell Biologics Inc. (no. C57-6023; Chicago, IL, USA). The BMECs were plated and cultured to a confluence of 90% before oxygen-glucose deprivation (OGD) treatment. OGD treatment was established as follows: cells were rinsed once with glucose-free DMEM (Gibco, Rockville, MD) and transferred to an anaerobic chamber (Forma Scientific, Waltham, MA) containing a gas mixture composed of 7% CO_2_ and 93% N_2_ for 6 h at 37°C. Then, the cells were returned to the normal culture condition. Control BMECs were cultured in complete DMEM under normal conditions. AS-IV, HSYA, HSYA + AS-IV, and HQI + HHI groups were treated with AS-IV, HSYA, HSYA + AS-IV, and HQI + HHI (dissolved in complete DMEM and filtered with a 0.22 *μ*m membrane filter) for 12 h respectively after OGD treatment. Huang Qi injection (HQI) was purchased from Qingchunba (Chiatai, China) and Honghua injection (HHI) was purchased from Sanjiu (Ya'an, China). AS-IV and HSYA were purchased from the National Institute for Control of pharmaceutical and Biological Products. The groups and drug concentrations are listed in Table 1.

### 2.4. Cell Viability and Proliferation Analysis

For cell counting kit-8 (CCK-8) assays, experiments were performed in 96-well plates. Posttreatment, CCK-8 assays were performed in accordance with the manufacturer's instructions (MCE, HY-K0301; Shanghai, China), and readings were measured with a microplate reader from Thermo Scientific (Fremont, CA, USA). Cell proliferation was assessed using the BrdU cell proliferation assay kit from Biovision (Milpitas, CA, USA). Briefly, cells were plated in 12-well plates. After all the treatments mentioned, BrdU labeling was performed in accordance with the manufacturer's instructions. Next, the cells were analyzed with a MoFlo Astrios cell sorter from Beckman-Coulter (CA, USA).

### 2.5. Wound Healing and Transwell Analysis

Wound healing and transwell analyses were performed as previously described [31]. Briefly, cells were plated in 6-well plates. A wound on the cell monolayer was created by scratching the layer with pipette tips. Images were taken immediately and 24 h after scratching. The wound-closure percentages were calculated using the following formula: 1  − current wound size/initial wound size × 100; Costar (Cambridge, MA, USA) was added into 24-well culture plates. All cells were starved overnight in FBS-free DMEM. The cells (1 × 10^5^) were next seeded in the upper chamber in 200 *μ*L DMEM, whereas the lower chamber was flooded with DMEM (10% FBS). After 18 h, the wells were fixed with 4% polyoxymethylene and stained with crystal violet.

### 2.6. Cell Adhesion and Tube Formation

Cell adhesion and tube formation experiments were performed as previously described [32]. After treatments, BMECs (1,000 cells) were added to 24-well plates coated with fibronectin. BMECs that had adhered to fibronectin were counted under a phase-contrast microscope. Experiments were performed in triplicate.

BMECs (100,000 cells per well) were seeded into 24-well plates (100,000 cells per well) coated with 200 *μ*L of growth factor-reduced Matrigel from BD Biosciences. Branches were quantified after a 24 h incubation at 37°C. Images were taken with the SteREO Discovery.V20 microscope from Carl Zeiss (Jena, Germany), equipped with a plan apochromat 1.0x and the AxioCam HRc digital microscope camera from Carl Zeiss, automated with the AxioVision Rel.4.8 software from Carl Zeiss. Experiments were performed in triplicate.

### 2.7. Cell Apoptosis

Terminal deoxynucleotidyl transferase dUTP nick end labeling (TUNEL) assays were performed using the TransDetect In Situ Fluorescein TUNEL Cell Apoptosis Detection Kit (FA201-02) (Beijing, China). Briefly, cells were plated onto cover glasses in 6-well plates. After treatments, TUNEL experiments were performed and images were taken with the SteREO Discovery.V20 microscope from Carl Zeiss (Jena, Germany), equipped with a plan apochromat 1.0x and the AxioCam HRc digital microscope camera from Carl Zeiss, automated with the AxioVision Rel.4.8 software (Carl Zeiss).

Cell apoptosis was also analyzed using the Annexin V-FITC Apoptosis Kit (K101) from Biovision (Milpitas, CA, USA), in accordance with the manufacturer's instructions. After labeling, the cells were analyzed using a MoFlo Astrios cell sorter from Beckman-Coulter.

### 2.8. Small Interfering RNA Knockdown

Cells were transfected using Lipofectamine 2000 (Invitrogen, USA) with small interfering RNAs (siRNAs) targeting PHLPP-1 (GenePharma, Shanghai, China). A scrambled siRNA was used as a negative control. The siRNA sequences used are as follows: siPHLPP-1-1, 5′-GCAAGUGCCAAACAGUUCUTT-3′; siPHLPP-1-2, 5′-ACAUUUGUAGAAUAUGGATT-3′; siPHLPP-1-3, 5′-CGGGAGGCCUCCAGUCUATT-3′; siScramble, 5′-UUCUCCGAACGUGUCACGUTT-3′ [33].

### 2.9. Protein Collection and Western Blotting

RIPA lysis buffer (60 *μ*L) from Solarbio (Beijing, China) was added to the wells of a 6-well plate. At least three independent experiments were performed. The cell lysate was centrifuged at 14,000 rpm for 15 min. The resultant supernatant was mixed with 2x SDS-loading buffer and then subjected to western blotting. The protein samples were analyzed using SDS-PAGE. Western blotting was performed using standard procedures, and immune-reactive proteins were visualized using the SuperSignal™ chemiluminescence kit from Thermo Scientific. Anti-BCL-2 antibody, anti-Bax antibody, anti-VEGF antibody, anti-VEGFR2 antibody, anti-eNOS antibody, and anti-p-eNOS antibody were purchased from CST (Woburn, USA), whereas anti-GAPDH antibody, anti-Caspase-3 antibody, anti-cleaved Caspase-3 antibody, anti-caveolin-1 antibody, anti-Akt, anti-p-Akt, anti-GSK3*β*, anti-p-GSK3*β*, anti-PHLPP-1, and anti-actin antibody were purchased from Santa Cruz Biotechnology (CA, USA).

### 2.10. Intracellular Calcium Level Measurement

Intracellular calcium levels were measured as previously described [34]. Briefly, the cells were incubated in DMEM containing 10 *μ*M fluo 3-AM for 30 min at room temperature in the dark. Next, excess fluo 3-AM was removed and the intracellular calcium level of BMECs receiving various treatments was measured using the MoFlo Astrios cell sorter from Beckman-Coulter at an excitation wavelength of 488 nm and an emission wavelength of 505 nm.

### 2.11. Establishment of PHLPP-1 Knockout BMECs

Plasmid vectors containing lentivirus CRISPRv2 (puro, catalog 52,961), psPAX2 (catalog 12,260), and pVSVg (catalog 8454) were used to knockout PHLPP-1 in BMECs using the CRISPR-Cas9 technology. PHLPP-1-specific single-guide RNA (sgRNA) was designed using the online tool CRISPR DESIGN (http://CRISPR.mit.edu), synthesized, and cloned into the lentiCRISPR v2. Lentiviruses were packaged in 293 T cells and transfected into BMECs. Puromycin was selected from monoclonal cell lines. Western blotting confirmed the construction of cell lines with PHLPP-1 knockout.

### 2.12. Statistical Analysis

The SPSS version 18.0 (IBM Corporation, USA) was used for statistical analysis. Data were generated from at least three replicates and student's *t*-test or one-way analysis of variance (ANOVA) followed by Tukey's post-hoc test was performed. Data are presented as mean ± standard deviation, and *p* < 0.05 was considered significant.

## 3. Results

### 3.1. AS-IV and HSYA Promote BMEC Proliferation

**Figure 1:**
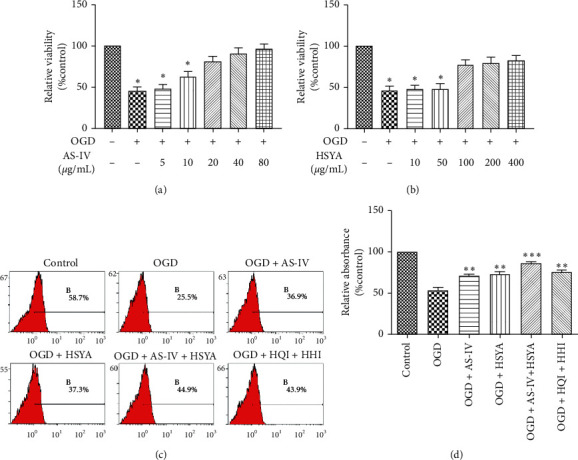
AS-IV and HSYA attenuate OGD-induced cell death and proliferation inhibition. (a) Histogram showing cell viability in the control, OGD, and OGD AS-IV groups; ^*∗*^*p* < 0.05 vs. the control group. (b) Histogram showing cell viability in the control, OGD, and OGD HSYA groups; ^*∗*^*p* < 0.05 vs. the control group. (c) Representative images of flow cytometry analyses of proliferation using BrdU staining in each group. All data are expressed as mean ± standard deviation (S.D.). (d) Histogram showing cell viability in the control, OGD, OGD + AS-IV, OGD + HSYA, OGD + HSYA + AS-IV, and OGD + HQI + HHI groups; ^*∗∗*^*p* < 0.001 and ^*∗∗∗*^*p* < 0.01 vs. the OGD group.

We first analyzed the contents of HQI (Huang Qi injection) and HHI (Honghua injection) using HPLC. In comparison with standard samples, our results showed that the concentration of AS-IV in HQI was 0.15 mg/mL and that of HSYA in HHI was 0.24 mg/mL (data not shown). To evaluate the functions of AS-IV and HSYA, we first established an *in vitro* ischemia-reperfusion model with BMECs. BMECs were cultured in glucose-free DMEM in a hypoxia incubator with a 95% N_2_ and 5% CO_2_ mixture for 2 h. After a 24 h recovery period, cell viability was determined with CCK-8 assays. Our results showed that the BMEC quantity was 50% less after OGD treatment as compared with the control group, suggesting that OGD treatment largely resulted in cell death (Figures 1(a)–1(c)). As expected, a supplement of AS-IV attenuated cell death. AS-IV (920 *μ*g/mL) rescued the cell viability (Figure 1(a)). HSYA (100 *μ*g/ml) rescued the cell viability, whereas 10 and 50 *μ*g/mL had little effects on cell viability (Figure 1(b)). In addition, treatment with AS-IV (20 *μ*g/mL) or HSYA (100 *μ*g/mL) after OGD significantly increased the viability, suggesting that both AS-IV and HSYA attenuated OGD-induced cell death (Figure 1(c)). Moreover, we found that the combination of HSYA and AS-IV (20 *μ*g/mL AS-IV + 100 *μ*g/mL HSYA) further rescued BMECs from the effects of OGD. For example, a combination of 2% HQI and 2% HHI (2 g/mL HQI + 0.5 g/mL HHI) significantly attenuated the OGD-induced decreased cell viability (Figure 1(c)). We further examined BMEC proliferation after treatment with various protectors through BrdU labeling, showing that the percentage of proliferating cells decreased from 58.7 to 25.5% after OGD (Figure 1(d)). However, the percentage of proliferating cells increased to 35.9% and 37.3% after treatment with AS-IV and HSYA, respectively. Treatment with a combination of AS-IV and HSYA further rescued proliferation (44.9%) (Figure 1(d)). For example, treatment with a combination of 2% HQI and 2% HHI increased the proliferation to 43.9%. These results suggested that AS-IV and HSYA attenuated the OGD-induced decrease in the proliferation.

### 3.2. AS-IV and HSYA Attenuate OGD-Induced Apoptosis

**Figure 2:**
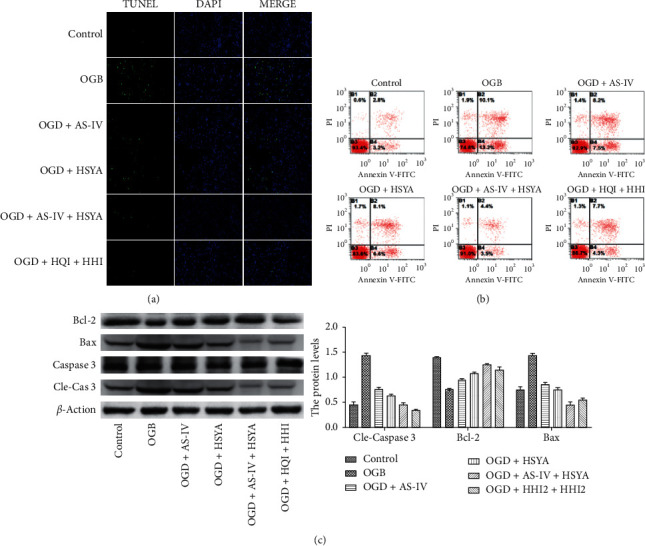
BMEC apoptosis inhibition by AS-IV and HSYA. (a) Representative images of TUNEL staining of cells. (b) Representative images of apoptosis in each group determined using flow cytometry. (c) Expression of apoptosis-related proteins Bax, Bcl-2, Caspase 3, Cle-Cas 3, and GAPDH assessed using western blotting. ^*∗*^*p* < 0.05 vs. the OGD group.

We next examined the OGD-induced BMEC apoptosis. As expected, the apoptosis of OGD BMECs significantly increased, as revealed by TUNEL staining (Figure 2(a)). However, BMEC culturing with AS-IV or HSYA significantly decreased apoptosis (Figure 2(a)). Moreover, the combination of AS-IV and HSYA provided a better protective effect against OGD (Figure 2(a)). Flow cytometry experiments further validated these results (Figure 2(b)). Thus, OGD reduced the viability by decreasing proliferation and increasing apoptosis, and AS-IV and HSYA attenuated this loss by increasing cell proliferation and inhibiting apoptosis. To further elucidate how AS-IV and HSYA inhibited BMEC apoptosis, we examined the expression of antiapoptotic gene Bcl-2 and proapoptotic gene Bax. Our results showed that OGD significantly induced the expression of Bax, Caspase 3, and cleaved Caspase 3 (cle-Cas 3) protein levels (Figure 2(c)). However, it downregulated the expression of the antiapoptotic gene *Bcl-2* (Figure 2(c)). Culturing OGD BMECs with AS-IV or HSYA attenuated the decreased expression of *Bcl-2* and decreased Bax, Caspase 3, and cle-Cas 3 levels (Figure 2(c)), indicating that AS-IV and HSYA inhibited OGD-induced BMEC apoptosis by directly regulating the expression of apoptosis-related genes.

### 3.3. AS-IV and HSYA Promote BMEC Migration and Invasion

**Figure 3:**
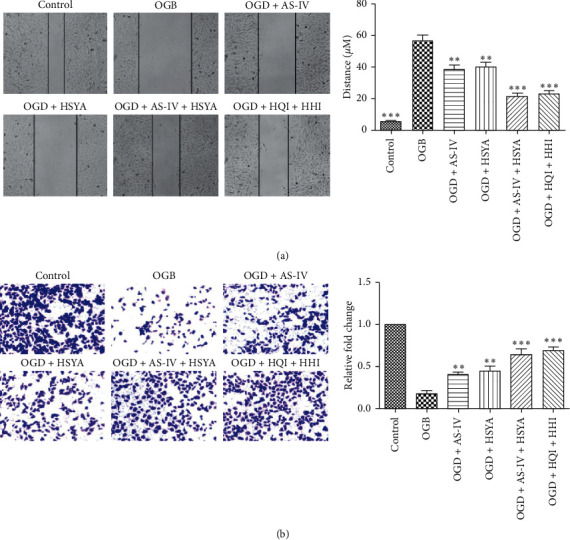
Promotion of BMEC migration and invasion by AS-IV and HSYA. (a) Representative images of a wound-healing assay and statistical analyses; ^*∗∗*^*p* < 0.01 and ^*∗∗∗*^*p* < 0.001 vs. the OGD group. (b) Results of transwell assay showing invasion of BMECs in each group and statistical analysis; ^*∗∗*^*p* < 0.01 and ^*∗∗∗*^*p* < 0.001 vs. the OGD group.

Because AS-IV and HSYA promoted the proliferation and inhibited the apoptosis of BMECs, we explored the functioning of rescued cells using cell migration and invasion assays. Results of a wound-healing assay showed that OGD significantly impaired the migration ability of BMECs. However, OGD BMECs cultured with AS-IV and/or HSYA showed partially restored migration ability (Figures 3(a) and 3(b)). In addition, BMEC invasion decreased significantlyafter OGD; however, it was rescued by supplementation of AS-IV and/or HSYA (Figures 3(c) and 3(d)).

### 3.4. AS-IV- and HSYA-Rescued BMECs Are Functionally Activated In Vitro

**Figure 4:**
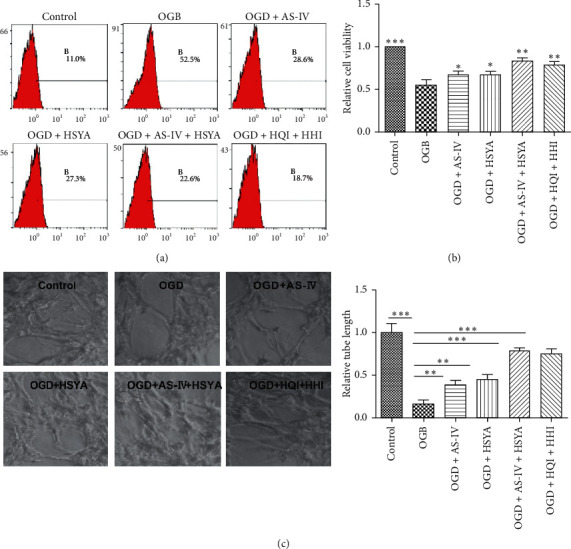
AS-IV and HSYA rescue of BMEC functional activity. (a) Intracellular Ca^2+^ concentrations measured using flow cytometry after cell incubation in fluo-3/AM. (b) Histogram showing cell adhesion; ^*∗*^*p* < 0.05 and ^*∗∗*^*p* < 0.01 vs. the OGD group. (c) Representative images of Matrigel tube formation and statistic analysis.; ^*∗∗*^*p* < 0.01 and ^*∗∗∗*^*p* < 0.001 vs. the OGD group.

During the stroke, BMECs or leucocytes adhere to the vessel wall to allow binding of activated platelets to the wall of inflamed microvessels [35, 36]. Thus, we examined the adhesion ability of AS-IV- and/or HSYA-rescued BMECs by plating them in wells coated with fibronectin. The results showed that OGD severely impaired the adhesion ability of BMECs, which was significantly attenuated by supplementation with AS-IV and/or HSYA (Figure 4(a)), suggesting that AS-IV- and/or HSYA-rescued BMECs were functionally activated.

The transendothelial, electrically resistant BBB is associated with intracellular calcium in the brain microvascular endothelial cells *in vitro* [34, 37]. However, AS-IV inhibits spontaneous synaptic transmission and synchronized Ca^2+^ oscillations in neuronal cells [38]. We assessed the intracellular Ca^2+^ concentration of fluo 3-AM-treated BMECs via flow cytometry. The results showed a significant increase in the intracellular Ca^2+^ concentration of OGD BMECs, which was attenuated by treatment with AS-IV and/or HSYA, suggesting the protective function of both AS-IV and HSYA in ischemia-induced BBB damage (Figure 4(b)).

To further explore the role of AS-IV and HSYA in ischemia-reperfusion injury, we plated BMECs on Matrigel and evaluated tube formation *in vitro*. Our results demonstrated that OGD significantly decreased the tube formation as the tube length of OGD BMECs decreased to approximately 20% of that of the control BMECs (Figures 4(c) and 4(d)). However, OGD BMECs cultured with AS-IV and/or HSYA showed significantly recovered tube formation, with their combination exerting a synergistic effect (Figures 4(c) and 4(d)).

### 3.5. AS-IV and HSYA Protect BMECs via Regulating Multiple Signaling Pathways

**Figure 5:**
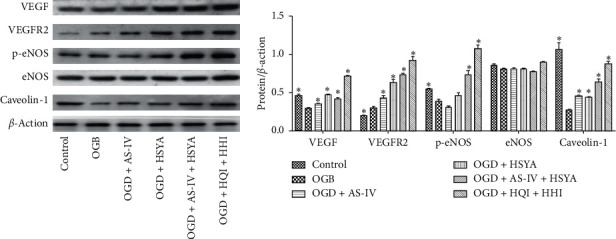
AS-IV and HSYA regulation of VEGF and eNOS signaling. Western blot analyses of the expression of VEGF, VEGFR2, P-eNOS, eNOS, caveolin-1, and ACTIN. ^*∗*^*p* < 0.05 vs. the OGD group.

Because tube formation in OGD BMECs was rescued by AS-IV and HSYA, we examined their association with vascular endothelial growth factor (VEGF) signaling. VEGF and VEGF receptor 2 (VEGFR 2) levels were found to be significantly upregulated following treatment with AS-IV and/or HSYA, despite their unaltered levels in OGD BMECs (Figure 5).

In BMECs, nitric oxide (NO) can be derived from intracellular endothelial NO (eNOS), which serves as a hypoxia signal in these cells [39]. Increased phosphorylated eNOS (p-eNOS) levels subsequently activated the antioxidant signaling, which protected the BMECs [40]. Interestingly, our results indicate that eNOS levels in BMECs were unaltered although treatment with AS-IV and/or HSYA significantly increased the levels of p-eNOS, activating antioxidant signaling in BMECs (Figure 5).

The caveolin pathway plays a critical role in preserving the integrity of BBB [41]. Our results showed that OGD significantly decreased caveolin-1 protein levels, which was significantly attenuated by treatment with AS-IV and/or HSYA, suggesting that AS-IV and HSYA protected BMECs by stimulating the caveolin-1 pathway (Figure 5).

**Figure 6:**
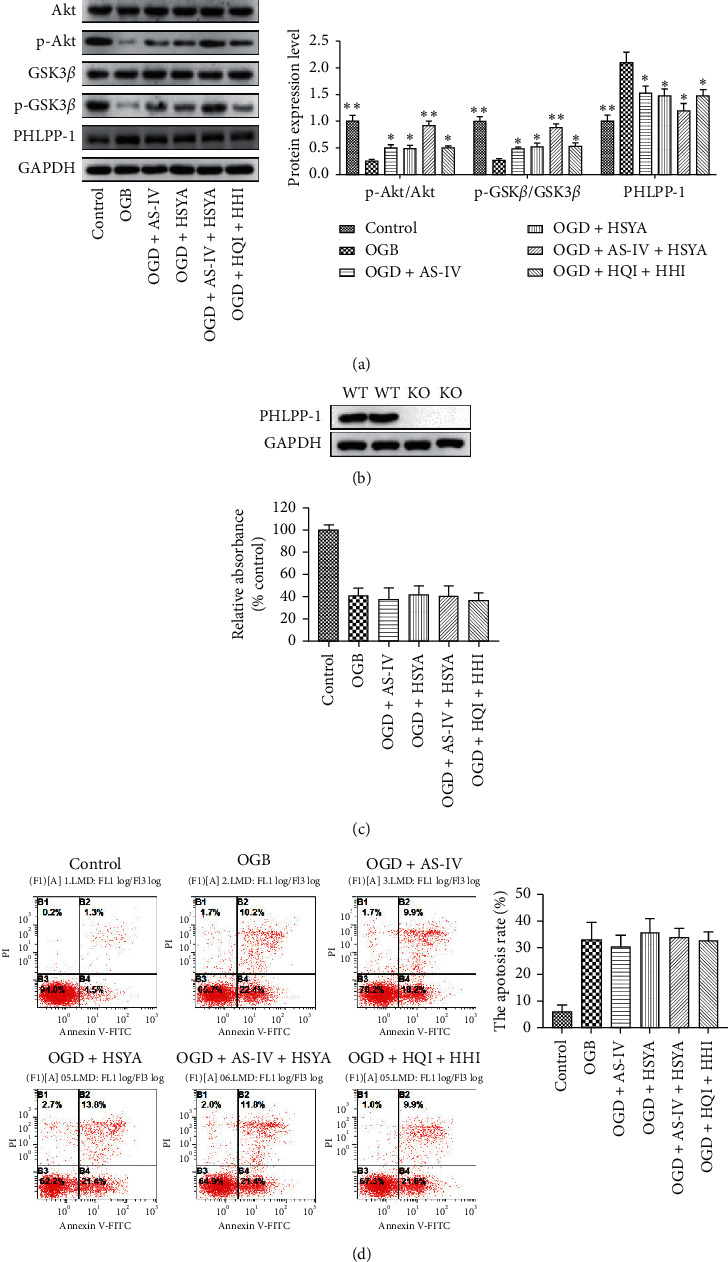
AS-IV and HSYA regulate Akt signaling via PHLPP-1. (a) Western blot analyses of the expression of Akt, phosphorylated-Akt, GSK3*β*, phosphorylated GSK3*β*, PHLPP-1, and GAPDH; ^*∗*^*p* < 0.05 and ^*∗∗*^*p* < 0.01 vs. the OGD group. (b) Western blot analyses of the expression of PHLPP-1 and GAPDH in control or PHLPP-1 knockout cells. (c) Histogram showing cell viability in the control, OGD, and OGD AS-IV groups with PHLPP-1 knockout. (d) Histogram showing cell apoptosis in the control, OGD, and OGD AS-IV groups with PHLPP-1 knockout.

We further analyzed the potential molecular alteration caused by AS-IV and HSYA treatment and found that AS-IV and/or HSYA treatment led to increased levels of phosphorylated Akt and phosphorylated GSK3*β*, but not total Akt or GSK3*β*, which were significantly increased in comparison to the OGD treatment-alone group (Figure 6(a)). In addition, HHI and HQI treatment showed identical effects (Figure 6(a)) suggesting the involvement of Akt signaling. Recently, reports showed that PHLPP-1 as a novel phosphatase negatively regulates the Akt activity [42–44]. Hence, we analyzed the expression of PHLPP-1 in AS-IV and/or HSYA-treated OGD. Surprisingly, our results showed that PHLPP-1 levels increased following OGD treatment and decreased following AS-IV and/or HSYA treatment (Figure 6(a)). Thus, we established PHLPP-1-knockout BMECs (Figure 6(b)) to further study the role PHLPP-1 plays in AS-IV and HSYA protection. Importantly, we found that the protective effects of AS-IV, HSYA, HQI, and HHI in PHLPP-1 knockout BMECs were attenuated as revealed by cell viability and apoptosis (Figures 6(c) and 6(d)). These data further suggested that AS-IV and HSYA-mediated ischemia-reperfusion protection was dependent on PHLPP-1/Akt.

## 4. Discussion

Several traditional Chinese medicines have been demonstrated to inhibit ischemia-induced excitotoxicity and apoptosis and promote proliferation and angiogenesis [45]. Among these, AS-IV protects against ischemia-reperfusion injury, as demonstrated using several *in vivo* and *in vitro* models [18, 46, 47]. Results from the present study showed that AS-IV treatment significantly promoted BMEC proliferation *in vitro*, which is in agreement with the findings of a previous study in mesenchymal stem cell-derived endothelial cell-like cells that showed AS-IV-induced endothelial cell-like cell proliferation [48]. We found that AS-IV inhibited BMEC apoptosis by upregulating the expression of BCL2 and downregulating the expression of Bax, which is in line with the findings from a previous study in rats that showed AS-IV-inhibited cell apoptosis via TLR-4 signaling *in vivo* [14]. Thus, AS-IV exerts dual protective effects in BMECs *in vitro*; it increases proliferation and decreases cell death. HSYA was found to have a proproliferation role, as it inhibited ischemia-reperfusion injury-induced apoptosis [26, 27, 49]. Results from the present study demonstrate that this process is at least partially dependent on BCL2/Bax because HSYA treatment significantly attenuated the OGD-induced BCL2 reduction and Bax stimulation. Cotreatment with AS-IV and HSYA ameliorated cerebral infarction with Qi deficiency and blood stasis syndrome [50]. It was recently shown that Buyang Huanwu decoction, containing both AS-IV and HSYA, ameliorated the effects of ischemic stroke [51]. Our study results demonstrate that the combination of AS-IV and HSYA synergistically promoted proliferation and inhibited apoptosis, elucidating their benefits in ischemia-reperfusion injury. Further elucidation of the pathology of stroke, similar to the illumination of that of ischemia-reperfusion injury after induction of acute myocardial infarction [52], is required to facilitate a more precise use of these traditional Chinese medicines.

To validate the functioning of AS-IV and/or HSYA in rescuing BMECs *in vitro*, we first addressed both the migration and invasion abilities of AS-IV- and HSYA-treated BMECs and found significant improvement when compared with the OGD group. In addition, the synergistic migration and invasion ability increasing the effect of cotreatment with AS-IV and HSYA was stronger than the synergetic proliferation promotion and apoptosis inhibition effect, suggesting that the rescued BMECs were functioning. Furthermore, previous studies have shown that AS-IV and HSYA promoted migration and invasion [53, 54]. Moreover, our results demonstrated that the impaired cell adhesion ability, intracellular calcium levels, and tube formation in OGD BMECs *in vitro* were attenuated by treatment with AS-IV and/or HSYA, further validating the functional activation of AS-IV- and/or HSYA-rescued BMECs.

AS-IV and HSYA protect the central nervous system via multiple mechanisms. Our study demonstrated that VEGF signaling was activated in ODG BMECs treated with AS-IV and HSYA, explaining the attenuated tube formation ability. VEGF signaling is tightly regulated in BMECs. This, in turn, regulates BMECs, alleviating BBB disruption, cerebral edema, and neuronal injury [10, 55, 56]. Furthermore, we observed significantly increased P-eNOS levels rather than eNOS levels in OGD BMECs treated with AS-IV and/or HSYA, suggesting that AS-IV and/or HSYA treatment facilitates stroke recovery by modulating endothelial nitric oxide synthase. In addition, it was reported that caveolin signaling is one of the downstream targets of P-eNOS [41]. Our results showed that OGD significantly reduced the protein levels of caveolin-1, which was ameliorated by AS-IV and/or HSYA treatment. More importantly, our data revealed that AS-IV and HSYA mediated ischemia-reperfusion protection in a PHLPP-1/Akt-dependent manner. However, a detailed mechanism of how AS-IV and HSYA modulate the expression of PHLPP-1 requires further investigations. It is of special significance to check this protective mechanism of AS-IV and/or HSYA treatment in myocardial ischemia-reperfusion because PHLPP-1 plays crucial roles in myocardial ischemia-reperfusion [30, 43, 44].

## 5. Conclusions

Treatment with AS-IV and/or HSYA protected BMECs against ischemia-reperfusion injury by promoting proliferation and inhibiting apoptosis *in vitro*. In addition, it rescued the functioning of BMECs. Treatment with AS-IV and/or HSYA protected BMECs against ischemia-reperfusion injury by stimulating VEGF and eNOS signaling. These results further illustrate the benefits of AS-IV and HSYA in stroke prevention and treatment, which may not only be advantageous in stroke cases but other ischemia-reperfusion injury cases.

## Figures and Tables

**Table 1:** Drug concentrations of each group indicated are as follows (if not specified in the figures).

Group	Treatment
Control	Same volume of PBS
OGD	Same volume of PBS
AS-IV	20 *μ*g/ml astragaloside diluted from HQI
HSYA	100 *μ*g/ml hydroxysafflor yellow A diluted from HHI
AS-IV + HSYA	20 *μ*g/ml astragaloside diluted from HQI and 100 *μ*g/ml hydroxysafflor yellow A diluted from HHI
HQI + HHI	2% HQI (2 g/ml AS-IV) + 2% HHI (0.5 g/ml HSYA)

## Data Availability

The data used to support the findings of this study are included within the article.

## Abbreviations

AS-IV: Astragaloside
HSYA: Hydroxysafflor yellow A
BMEC: Brain microvascular endothelial cells
OGD: Oxygen-glucose deprivation
VEGF: Vascular endothelial growth factor
BBB: Blood-brain barrier.
